# Application of Chromatographic and Spectroscopic Methods towards the Quality Assessment of Ginger (*Zingiber officinale*) Rhizomes from Ecological Plantations

**DOI:** 10.3390/ijms18020452

**Published:** 2017-02-20

**Authors:** Wojciech Koch, Wirginia Kukula-Koch, Zbigniew Marzec, Elwira Kasperek, Lucyna Wyszogrodzka-Koma, Wojciech Szwerc, Yoshinori Asakawa

**Affiliations:** 1 Chair and Department of Food and Nutrition, Medical University in Lublin, 4a, Chodźki Str., 20-093 Lublin, Poland; kochw@interia.pl (W.K.); zbigniew.marzec@umlub.pl (Z.M.); elwira.kasperek7@gmail.com (E.K.); wyszogrodzka@op.pl (L.W.-K.); 2 Chair and Department of Pharmacognosy with Medicinal Plant Unit, Medical University in Lublin, 1, Chodzki str., 20-093 Lublin, Poland; 3 Department of Analytical Chemistry, Medical University in Lublin, 4a, Chodźki Str., 20-093 Lublin, Poland; wojciech.szwerc@umlub.pl; 4 Department of Pharmaceutical Chemistry, Faculty of Pharmaceutical Sciences, Tokushima Bunri University, Yamashiro-cho, Tokushima 770-8514, Japan; asakawa@ph.bunri-u.ac.jp

**Keywords:** *Zingiber officinale*, elements, terpenes, LC-MS, GC-MS, phenolic compounds

## Abstract

The usefulness of ginger in the food industry and pharmacotherapy is strictly related to its content of various components. The study elucidates the chemical composition of *Zingiber officinale* rhizomes cultivated on ecological plantations on Shikoku Island (Japan). GC-MS analysis of terpene content, LC-MS determination of phenolic content, and the determination of 12 elements using AAS spectrometry were performed to give more detailed insight into the samples. Ninety-five percent of terpene composition was elucidated, with zingiberene as the most abundant sesquiterpene (37.9%); the quantification of gingerols and shogaols was performed, showing the highest contribution of 6-gingerol (268.3 mg/kg); a significant K (43,963 mg/kg of dry mass) and Mn (758.4 mg/kg of dry mass) content was determined in the elemental analysis of the rhizomes and low concentration of toxic elements (Cd, Ni and Pb) remaining below the safe level values recommended by European Commission Directives. The main phenolic compound was (6)-gingerol, which is characteristic of fresh rhizomes and is responsible for their taste and aroma. Surprisingly, high amounts of (6)-shogaol were determined, even though this phenolic compound usually occurs in old or processed material and not in fresh rhizomes. Sesquiterpenes were the major fraction of volatiles. The highest concentrations were determined for α-zingiberene, β-sesquiphellandrene, (*E*,*E*)-α-farnesene, geranial, and *ar*-curcumene. The volatiles composition of ginger cultivated on Shikoku Island is specific and strongly differs from plants cultivated in China, Nigeria, or Australia. The elemental composition of ginger rhizomes grown in ecological plantations is more beneficial for human health compared to products grown in normal cultivars, as the products contain high amounts of potassium and manganese and are characterized by low sodium content and lower levels of toxic heavy metals.

## 1. Introduction

Interest in nutrition, natural healing methods, and the use of medicinal plants is increasing every year. One of the most important plants used in both medicine and cuisine is *Zingiber officinale* Roscoe. The use of ginger in traditional medicine has been reported since antiquity, as early as 3500 BC [1]. Ginger belongs to the family Zingiberaceae and is cultivated in various countries and around the world [2]. Ginger is now one of the most important and popular spices on the international market and the total global production of ginger is estimated at 100,000 tons a year. All over the world, the ginger rhizome is appreciated for its taste qualities; therefore, it is used as a spice, flavoring agent, and as an additive in the preparation of meals [3]. It is also used as an ingredient in dietary supplements such as pills, syrups, or teas. The ginger rhizome or its extracts have been commonly used in medicine, because of their wide scope of biological effects—confirmed both in various in vitro models and in clinical trials. The plant has been found to show strong antiemetic activity and is now used to treat motion sickness, morning sickness, and post-chemotherapy nausea [4]. Moreover, its analgesic and painkilling properties have been applied in pharmacotherapeutical strategies in the treatment of osteoarthritis due to the marked anti-inflammatory properties of the plant. The confirmed antimicrobial action of the rhizomes (including antibacterial, antiviral, antifungal, and antiparasitic activity) justify its traditional use in cold treatment [5,6,7]. The active compounds from ginger rhizomes inhibit platelet aggregation and have a strong vasodilatory effect, which decreases blood pressure and improves blood circulation [8]. Some studies suggest that ginger may be a potential drug in the treatment of diabetes and hypercholesterolemia because of its hypoglycemic and hypolipidemic properties [9,10]. Many reports provide information on ginger antineoplastic properties, which refer to its antioxidant, anti-inflammatory, and anti-mutagenic effects and the ability to intensify tumor cell apoptosis. Moreover, studies have confirmed ginger properties in the treatment of skin, breast, brain, or liver cancer [11]. The two major groups of active compounds from ginger, which are responsible for most of the biological actions of this plant, are polyphenols (gingerols, shogaols, and paradols) and volatiles such as zingiberole, zingiberone, and zingiberene [12].

Plants are important sources of elements, both those essential for humans and also toxic ones such as cadmium, lead, or nickel. The elemental composition of a plant should be considered as a whole. The content of elements is not the only important factor; it is important to note their proportions and the forms the elements exist in. The elemental composition can be influenced by many factors, e.g., the origin of the raw material, the time of collection, the geochemical conditions of the area, and environmental pollution. The World Health Organization emphasizes that the content of elements should be controlled, particularly the content of heavy metals in medical plants, to maintain their purity, quality, and safe usage [13].

The wide-ranging pharmacological applications of ginger and the increasing consumption of the spice encouraged the authors to perform these studies on the chemical composition of ginger rhizomes, as there are no data in the literature on the active components or element content of ginger grown on Shikoku Island. Ginger cultivars from different regions vary significantly in both elemental and metabolite composition. Based on the above information, the authors established a hypothesis that ginger cultivated on ecological plantations will be characterized by higher pharmacological and nutritional value and lower levels of toxic elements compared to plants from routine cultivars. Therefore, the second goal of the paper is to determine if ginger cultivated on ecological plantations in Japan contains higher amounts of macro- and trace elements important in human nutrition, and lower levels of heavy metals (cadmium, lead, and nickel) that are harmful to humans.

To the authors’ knowledge, this is the first wide study on the chemical composition of ginger rhizomes grown in ecological plantations using fully validated chromatographic and spectroscopic methods. Our study revealed several important differences in chemical composition between gingers obtained from ecological plantations and in the traditional way.

## 2. Results and Discussion

Ginger rhizomes have been extensively used in medicine for many centuries. However, as previously reported [14,15,16], their composition varies markedly depending on the geographical area of origin. According to Sasikumar and co-investigators [17], there are more than 50 cultivars of this plant species in India alone. That is why the variations between the tested samples, often from distant locations, are significant.

The constituents of ginger’s rhizome may be divided into two major groups: lipids and oleoresin. The latter is composed of terpenes (monoterpenes, sesquiterpenes, and sesquiterpene alcohols) comprising 20%–25% and phenolics–gingerols (including gingerodiones, gingersiols, dehydrogingerdiones, and diarylheptanoids), which account for another 25% of the semi-liquid resin from the rhizomes [18,19].

Japanese ginger studied in the presented study was derived from two famous ecological plantation areas in the country, Kochi and Tokushima, located on the island of Shikoku. The aim of this paper was to fully characterize the cultivars from Japan. In view of this hypothesis, GC-MS based determination of terpenes, LC-MS analysis of phenolics, and AAS quantification of bioelements and heavy metals in this pharmacologically precious plant material were performed.

### 2.1. Qualitative and Quantitative GC-MS and LC-MS Analysis of Extracts

#### 2.1.1. GC-MS Profiling of Diethyl Extracts

The spectrometric analyzes showed the presence of monoterpenes (such as α-pinene, camphene, myrcene, and α-phellandrene), oxygenated monoterpenes (geranial, citronellal, neral, linalool, borneol, and α-terpineol), and sesquiterpenes (α-and β-farnesene, *ar*-curcumene, zingiberene, zingiberenol, copaene, or cadinene) in the studied extracts. All identified compounds (listed in Table 1) represented ca. 95% of the total extract from ginger rhizomes. The most abundant substances in the extracts were α-zingiberene (37.9%), β-sesquiphellandrene (11.4%), (*E*,*E*)-α-farnesene (9.6%), geranial (8.2%), *ar*-curcumene (6.3%), and γ-terpinene (5.1%).

Japanese ginger is known from its high pharmacological potential and nutritional value and, as such, it is widespread in the Asian market. These results are consistent with the scientific literature; however, the contribution of terpenes to the total sum of terpenes differs between samples of different origin, as previously expected. The variations in the oil composition between different species of the plant may occur due to the unequal agroclimatic conditions in which ginger is cultivated or due to a diversity of ginger varieties [20]. According to Gupta and co-workers [20], north Indian samples of ginger rhizomes showed a significantly higher content of monoterpenes responsible for the smell of the spice, and a much lower concentration of sesquiterpenes (6.0%) and oxygenated sesquiterpenes (7.2%) in comparison with other cultivars. The relative percentage content of geraniol in these samples reached 14.5%, 9.5% geranial, 5.6% borneol, and 8.1% neral. Also, elevated monoterpene composition has been found to be characteristic of Madagascar cultivars [20]. On the other hand, south Indian rhizomes delivered mainly oxygenated sesquiterpenes, which constituted ca. 68% of the total oil composition and only ca. 13% of monoterpenoids [21]. However, the percentage of zingiberene, characteristic of ginger rhizomes, remained much lower in the described studies than in Japanese ginger and was around 27% in Indian cultivars [22], which is ca. 10% less than in the studied plant material.

The rhizomes cultivated in Nigeria have been described as potent sources of sesquiterpenes; however, the percentage of several major components differed from Japanese ginger, too. African cultivars contained a high percentage of *Z*-γ-bisabolene (12.5%) and twice as much *ar*-curcumene (11.3%), but the quantity of zingiberene was ca. 10% lower than in Japanese ginger [23].

Also, the scientific literature delivers information on the quality of the volatile composition in Chinese rhizomes, which seem to be the most similar to the Japanese cultivars. According to Yang and co-investigators [24], Chinese ginger contains the highest quantity of sesquiterpenes in their composition, resembling the material studied in this paper. Zingiberene, β-bisabolene, and β-sesquiphellandrene constitute ca. 39.0%, 5.9%, and 12.3%, respectively, similar to Japanese ginger, whereas the contents of *ar*-curcumene and (*E*,*E*)-α-farnesene are slightly lower (4.7% and 7.6%). The qualitative and quantitative compositions of monoterpenes in Chinese ginger vary from the studied samples. According to Yang and co-investigators [24], no geraniol or geranial were reported in their plant material, whereas in the Japanese samples these compounds accounted for more than 8.5% of the composition.

In sum, Japanese ginger has been proven to contain a high percentage of sesquiterpenes in its terpene fraction, with α-zingiberene, β-sesquiphellandrene, (*E*,*E*)-α-farnesene, geranial, and *ar*-curcumene as the major constituents. In comparison with other previously studied rhizomes cultivated in different areas of the world, Japanese samples of *Zingiber officinale* may be perceived as rich sources of zingiberene—a strong pesticide [25], geranial—an anti-inflammatory agent [26], and (*E*,*E*)-α-farnesene, known for its antibacterial and antifungal properties [27].

#### 2.1.2. LC-MS Profiling of Ethanolic Extract from Ginger Rhizomes

**Figure 1:**
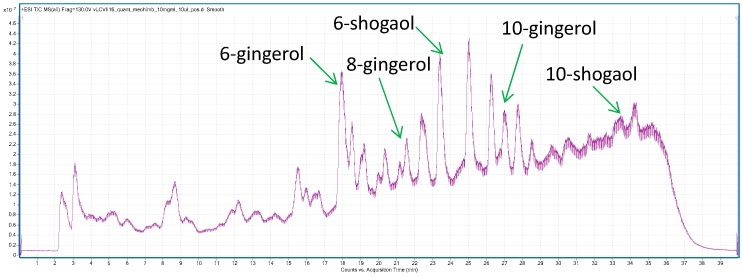
TIC spectrum of *Zingiber officinale* rhizome ethanolic extract with the analyzed phenolic compounds marked in accordance with their retention times.

Spectrometric analysis of the obtained ethanolic extracts revealed the presence of the five major phenolic compounds in ginger rhizomes (6)-, (8)-, and (10)-gingerols and (6)- and (10)-shogaols (Table 2, Figure 1).

Thanks to the application of an optimized LC method, all major phenolic constituents were well separated on a chromatographic column, which gave good MS/MS spectra. The fragmentation patterns of all the phenolic compounds listed above were carefully compared with those presented in the scientific literature and are presented in the (Figure S1) under the CID (collision induced dissociation) energy of 10 V. The application of higher collision energies (e.g., 20 V) in the LC-ESI-Q-TOF-MS analysis provided a lower intensity of MS/MS fragments and the loss of the molecular ion peak, which made the identification of compounds more difficult. Based on these observations, the 10 V CID energy value was selected as optimal for handling ginger phenolics.

Furthermore, the positive mode of operation was found under the set conditions, as it delivered higher sensitivity data in comparison with the negative mode. This observation was made after the analysis of two reference compounds in the applied conditions, 6-shogaol and 6-gingerol. Both compounds delivered ca. 2.5 times larger peak areas in the positive mode, compared to negative mode settings at the same concentrations.

The quantitative analysis of the five selected phenolic compounds was performed based on the calculated calibration curve of 6-gingerol (for all studied gingerols) and 6-shogaol (for both shogaols).

The calibration curve equations, *R*^2^ values, LOD (limits of detection), and LOQ (limits of quantification) values for both reference compounds were as follows: *y* = 101014292*x* − 58116266; *R*^2^ = 0.9932; 0.65 ng/mL for (6)-gingerol and 1.94 ng/mL and *y* = 89336828*x* − 56651211; *R*^2^ = 0.997; 0.75 ng/mL and 2.24 ng/mL for (6)-shogaol. The results were obtained after a 5-fold injection of standards with an injection volume of 20 µL each. The LOQ was determined as a triple value of the obtained LOD.

The major compound present in the highest amount was (6)-gingerol, which is in accordance with the literature [28,29]. This phenol is characteristic of fresh rhizomes and is responsible for ginger’s characteristic taste and aroma; it is more pungent than (8)-gingerol or (10)-gingerol [18]. Although the plant material was fresh, the content of (6)-shogaol, which normally occurs during dehydratation of gingerols and is characteristic for old or processed rhizomes, was significantly high [30]. The high content of this compound in fresh Japanese ginger may be responsible for its strong, spicy taste. The content of (6)-gingerol was higher compared to that of different cultivars of fresh Australian ginger determined by Wohlmuth et al. [14]. However, the concentrations of (8)- and (10)-gingerols were at a similar level. Therefore, due to the high content of (6)-gingerol, the mean ratio of (6)-gingerol/(8)-gingerol/(10)-gingerol was close to 4:1:1, which was higher than that found in 17 varieties of ginger cultivated in Australia [14]. The large concentration of (6)-shogaol may also show important health benefits, as this compound has been shown to have strong anticancer activity. Therefore, some authors suggest that steamed ginger has more significant anticancer properties than fresh plant material [31]. Our study elucidated that fresh ginger cultivated on ecological plantations in Shikoku Island contains high amounts of both (6)-gingerol and (6)-shogaol.

### 2.2. Macroelement Content in Ginger Samples

The content of 12 metals was determined in the samples of ginger rhizome cultivated on Shikoku Island (see Table 3).

Of all the studied macroelements, the highest concentration was measured for potassium and the lowest for sodium. These results are consistent with the data presented in other studies of ginger grown in different parts of the world, which have revealed that ginger is a good source of potassium but has low sodium content [13,32,33]. It should be emphasized that the outcomes of these analyses confirmed a much higher content of both potassium and sodium in the Japanese samples compared to other studies. Pandotra et al. [33] studied the elemental composition of ginger rhizomes from different areas of India and obtained an average content of 6389 and 275 mg/kg DM for potassium and sodium, respectively. Much higher amounts of these macroelements have been confirmed in the ginger rhizomes cultivated in Nigeria—25,280 and 322 mg/kg DM for potassium and sodium, respectively. However, these values are still much lower than those of the Japanese ginger samples studied here [13]. Because of its high concentration of potassium, the fresh rhizome can be considered an important source of this element in the human diet, as a portion of only 20 g (from all sources) can deliver more than 80 mg of this mineral. Similarly, the magnesium concentration in Japanese samples exceeded that presented in other scientific papers. Indian samples have been described to contain 2616 mg/kg DM—much less than that described here; however, African samples have been found to be richer in magnesium: Nigerian = 4210 mg/kg DM or Ethiopian = 4890 mg/kg DM [13,33,34]. The content of calcium in ginger from different parts of the world undergoes some variations. In our study, we obtained much higher results for its quantitative measurements compared to the average content in ginger cultivated in India (304 mg/kg DM), but these results were comparable to ginger grown in Ethiopia (2000–2540 mg/kg DM) [33,34]. Even though the content of calcium or magnesium in the rhizome turned out to be high (1587 mg/kg DM), considering an average daily consumption of fresh ginger of 20 g, this plant cannot be considered a good source of calcium and magnesium in the diet.

### 2.3. Trace Element Content of Ginger Rhizomes

Among microelements, manganese was found to be the leading constituent of the rhizomes. Its average content was significantly higher than in the results obtained from Nigerian (0.02 mg/kg DM) and Indian samples (43.3 mg/kg DM), but lower compared to ginger cultivated in Ethiopia, which contained 184–401 mg/kg DM [32,33,34]. The main sources of manganese for humans are plant products such as vegetables and grains [35]. Because of the high content of manganese in ginger, one portion of this plant contains over 60% of the recommended dietary allowance for this element, estimated at 2.3 mg for men and 1.8 mg for women. Therefore, the raw rhizome of ginger cultivated in Shikoku could be an important source of this element in the human diet [36]. Moreover, the studied ginger was rich in iron (12.5 mg/kg in fresh rhizome and 141 mg/kg DM), copper (1.03 mg/kg in fresh rhizome and 11.1 mg/kg DM), and zinc (2.5 mg/kg in fresh rhizome and 25 mg/kg DM). The content of these elements is different in different regions of the world. Rhizomes from India contain higher amounts of iron but significantly lower contents of zinc and copper. The content of iron in the ginger cultivated in Nigeria and Ethiopia is comparable to ginger from Shikoku, but the content of zinc and copper is lower [32,33,34]. Even though the content of iron, copper, and zinc in the studied material is high, ginger cultivated in Shikoku Island cannot be considered an important source of these elements in the human diet. Moreover, small amounts of chromium were determined in the investigated material: 0.064 mg/kg in fresh rhizome and 0.795 mg/kg DM. Amounts determined in ginger from India (0.88 mg/kg DM) and Algeria (0.57 mg/kg DM) are at a similar level, whereas a much higher concentration of chromium has been found in ginger cultivated in Ethiopia (6.02–10.8 mg/kg DM) [33,37]. Adequate intake (AI) of chromium has been established as 35 µg for men and 25 µg for women; therefore, the investigated ginger samples cannot be considered a good source of this element for humans [36].

### 2.4. Heavy Metal Content in Ginger Cultivated on Shikoku Island

Of the analyzed heavy metals, nickel had the highest content; however, its average concentration was significantly lower than in rhizomes of the same species from India (0.72 mg/kg DM) and Ethiopia (5.56–8.40 mg/kg DM) [34,35]. The determined level of nickel was far below the recommended value of 10 mg/kg established by the WHO [38]. Although, according to some studies, nickel can be an important cofactor of certain metalloenzymes and can participate in the absorption of iron, it is considered a contaminant and potentially hazardous element for humans and animals [38].

In the case of lead, the results are similar to the data reported for ginger cultivated in India (0.32 mg/kg DM) and China (0.39 mg/kg DM) [33,39]. In another study performed for ginger cultivated in different regions of India, the results varied over a large range from 0.06 to 0.6 mg/kg DM [20]. In ginger cultivated in Ethiopia and Nigeria, the concentration of lead is below the detection level [13,34]. Results from the present study and those obtained by other authors suggest that the concentration of metals in the soil has a significant influence on their content in different organs of the plant, especially in the roots or rhizomes. Therefore, the selection of the place from which the plants are collected may have an important influence on its biological and medicinal activity, including the health of the consumers. According to limits established by the European Committee in 2008, the concentration of lead in vegetables should not exceed 0.3 mg/kg [40]. Pb content in ginger investigated in the present study was far below this value.

Cadmium was the metal present in the lowest concentration in the investigated material. The average content of this element was far below the permissible level of 0.1 mg/kg established by the European Committee for root vegetables [40]. In the case of ginger from different regions of India, the cadmium concentration was much lower, while in the rhizomes grown in Nigeria this element was not detected [20,32,33]. However, results obtained in the present study were much lower compared to ginger cultivated in Ethiopia, which is characterized by a high cadmium content (0.38–0.97 mg/kg DM) [34]. Heavy metal content is related to the concentration not only in the soil, but also in chemical fertilizers, pesticides, and industrial equipment [41]. Therefore, the relatively high content of heavy metals in ginger from Shikoku Island could be related to the high level of industrialization of this region and the development of the iron and steel industry in Japan.

## 3. Materials and Methods

### 3.1. Reagents (Chemicals)

The standards of 6-gingerol and 10-shogaol were purchased from Sigma-Aldrich (St. Louis, MO, USA). Water, formic acid, and acetonitrile of spectroscopic grade were produced by J. T. Baker (Center Valley, PA, USA).

Nitric (V) acid (65%) and hydrochloric acid (36%) were of Suprapur Grade and were bought from Merck (Darmstadt, Germany). High purity deionized water (resistivity 18.2 MΩcm) obtained using an Ultrapure Millipore Direct-Q-R 3UV (Merck, Darmstadt, Germany) was used throughout the analysis. Quartz crucibles and polypropylene recipients were applied for the digestion and storage of digests, respectively.

A series of standard solutions of Ca, Cd, Cr, Cu, Fe, K, Mg, Mn, Na, Ni, Pb, and Zn at a concentration of 1000 mg/L (Merck, Germany) was used to plot the calibration curve for each element.

Diethyl ether and silica gel (230–400 mesh) applied in the GC-MS analysis of the extracts were produced by Merck (Germany). A C9–C25 hydrocarbon solution used as a reference mixture for GC-MS analysis was purchased from Sigma-Aldrich.

### 3.2. Investigated Plant Material

Raw rhizomes of ginger (*Zingiber officinale* Roscoe) cultivated in the ecological plantations on a Japanese island, Shikoku, were used in the present study. The investigated material was bought in Kochi and Tokushima over the course of six weeks in July and September 2013. Each week one series was purchased. The product was packed right after collection and stored at −22 °C.

### 3.3. GC-MS Analysis of the Rhizomes

#### 3.3.1. Sample Preparation

Ten grams of finely cut ginger rhizomes were moved to a mortar and ground together with the addition of ca. 10 mL of diethyl ether. The obtained extract was transferred to a silica-gel-filled Pasteur pipette. The obtained eluate (with water removed) was transferred to a vial and evaporated to dryness in the air. The procedure was repeated three times and the eluates joined in the same vial to provide high efficiency of extraction. The residue was resuspended in 1 mL of diethyl ether and used in the GC-MS analysis.

#### 3.3.2. The GC-MS-Based Study of Ginger Rhizomes

Ten microliters of the previously described diethyl ether extract were injected into a gas chromatograph (GC) (Agilent Technologies 4890N, Santa Clara, CA, USA) with a capillary column HP-5MS (30 m × 0.25 mm × 0.26) and an MS detector (Agilent Technologies 5973). The apparatus was operated in the mass range *m*/*z* of 40–500 in an EI mode at 70 eV, with a helium flow of 1 mL/min and inlet temperature of 250 °C. The temperature-dependent method was programmed in the following order: initial temperature = 50 °C, final temperature = 250 °C, and heating ramp = 5 °C/min. The initial temperature was held for 3 min and the final one for 15 min.

The identification of the extract’s constituents was performed in accordance with the scientific literature, the GC-MS libraries (Willey, NIST), the coinjected standards (the C9–C25 hydrocarbons solution co-injected with the sample in the similar conditions) and with respect to the obtained retention indices [42].

### 3.4. LC-MS Determination of Phenolics Present in Ginger Rhizomes

#### 3.4.1. Sample Preparation

Fresh ginger rhizomes were extracted using an ASE 100 apparatus (Dionex, Sunnyvale, CA, USA). For this reason ca. 5 g of plant material were placed in the extraction cell and then heated and extracted using 70% ethanol under the following conditions: static time—6 min, temperature—100 °C, pressure—ca. 114 bar, the number of cycles—three, and the cell volume—100%. The extracts were filled with 70% ethanol to a total volume of 50 mL and subjected to LC-MS analysis. From each batch of ginger six extracts were obtained.

#### 3.4.2. Determination of Phenolics in the Extract

The LC-Q-TOF-MS system, 6500 Series (Agilent Technologies, Santa Clara, CA, USA) equipped with an autosampler (G1329B), a degasser (G1322A), a DAD detector (G1315D), and a binary pump (G1312C) was used for chromatographic separation. A Q-TOF mass spectrometer (G6530B) was applied for the identification and determination of gingerols and shogaols in the obtained extracts. The injection volume was 20 µL of each standard and extract. The analytes were separated on a Zorbax RP-18 column from Agilent Technologies (dimensions: 150 mm × 2.1 mm, dp = 3.5 μm) in a flow rate of 0.2 mL·min^−1^ for 40 min. The mobile phase consisted of a combination of solvent A (0.1% formic acid) and solvent B (acetonitrile + 0.1% formic acid). The gradient elution was as follows: *t* = 0 min, 30% B; *t* = 10 min, 45% B; *t* = 15 min, 65% B; *t* = 25 min, 80% B; *t* = 30 min, 90% B; *t* = 31 min, 30% B. The photodiode detector continuously recorded the chromatograms in the range of absorbance from 190 to 500 nm. Mass spectra were simultaneously acquired using ESI in both negative and positive ionization modes with a capillary voltage of 4000 V. The mass spectra were recorded in the *m*/*z* range of 40 to 1500 *m*/*z*. The gas temperature and drying gas flow were 350 °C and 10 L·min^−1^, respectively, whereas those of sheath gas were 400 °C and 12 L·min^−1^. The skimmer and fragmentator voltages were set at 65 and 130 V, respectively. The nebulization pressure was 35 psig. The MS/MS spectra were recorded for the two most intensive peaks each time.

Three selected gingerols and two shogaols present in the extracts were determined qualitatively in the ethanolic rhizome extract based on their fragmentation spectra, the scientific literature, and retention times. A quantitative analysis of the selected phenolics was performed in accordance with the calibration curves of two standards. (6)-gingerol was used for the quantitative determination of all gingerols and (6)-shogaol for both shogaols. Also, the LOD and LOQ values of the optimized method were determined for both compounds.

### 3.5. The Elemental Analysis of the Samples

#### 3.5.1. Sample Preparation

From each batch six 25 g samples of ginger rhizome were cut into small fragments and weighed into pure quartz crucibles. Thirty-six samples were investigated in total. All of them were first dried in an electrical dryer in 105 °C for 24 h until a constant mass was obtained. At the same time, six samples of a reference material (10 g each) were weighted into quartz crucibles and applied to the same protocol as ginger samples. These samples did not require drying.

#### 3.5.2. Digestion of Samples

The analyzed samples (of the ginger and of the reference material) were digested by ashing at 450 °C in the muffle furnace. The ashing process was accelerated using a 30% water solution of nitric (V) acid, which was then evaporated. Later the samples were re-heated at 250 °C. The ash was dissolved in 10% hydrochloric acid and diluted with deionized water (1 + 1 *v*/*v*). The obtained solutions were transferred quantitatively into separate test-tubes, filtrated through filter paper to remove silica and other insoluble particles, and filled up to a volume of 25 mL with deionized water.

#### 3.5.3. Preparation of Standard Solution

To plot the calibration curve, standard solutions were performed for each element. Basic solutions of analyzed elements (Merck Standard Solution, concentration 1000 mg/L) were diluted 10-fold with deionized water and a working solution at the concentration of 100 mg/L was prepared. Through the dilution of proper quantity of working solutions, standard solutions of analyzed elements, were prepared (Table S1).

#### 3.5.4. Analytical Determination of Metals by FAAS and ETAAS

The determinations were performed using flame (FAAS) and electrothermal (ETAAS) atomic absorption spectrometry. The content of Ca, Cu, Fe, K, Mg, Mn, Na, and Zn was determined directly from the digests with F-AAS SOLAAR M5 apparatus (Thermo Scientific, Waltham, MA, USA) using, if needed, 10-, 500-, and 5000-fold dilutions. Lanthanum trichloride at a final concentration of 10% in the analyzed sample was used as a spectral buffer during calcium determinations.

The levels of Cd, Cr, Ni, and Pb were determined by electrothermal technique of atomic absorption spectrometry with atomization in a L’vov platform graphite cuvette using a High-Resolution Continuum Source Atomic Absorption Spectrometer ContrAA 700 (Analytik, Jena, Germany). Stock solutions at the concentration of 60 ppb (µg/L) for lead, 5 ppb for cadmium, 60 ppb for nickel, and 50 ppb for chromium were prepared by the dilution of standard solutions at a concentration of 1000 ppm (Merck) in 0.5% nitric acid, previously prepared by diluting the 65% Suprapur nitric acid (Merck) in deionized water. The calibration curves for the elements were selected by the decomposition of residues method. The dilution factor was chosen automatically by the autosampler. Twenty-five microliters of each sample solution with the addition of 5 µL of Pd(NO_3_)_2_/Mg(NO_3_)_2_ matrix modifier (when necessary) was injected into the furnace.

As a reference material used to validate the spectroscopic determination (AAS), a mixture of flour and milk powder (in proportions 70:30), fortified with known concentrations of investigated elements, was used. The content of investigated elements in the flour and milk mixture resulting from the fortification is presented in Table 4 and Table 5 as a theoretical value, whereas the quantities of the respective elements determined in the measurement are shown in the “found value” line. The analysis of the investigated and reference material were performed simultaneously under the same conditions.

Based on calibration curves, the obtained values were calculated to provide the content of elements in fresh ginger rhizome and in dry mass. Operating parameters for FAAS and ETAAS analysis are presented in the Tables S2 and S3. The limits of detection (LOD), as well as the recovery values, are presented in Table 4 and Table 5.

## 4. Conclusions

LC-MS analysis of the obtained ethanolic extract revealed that the main phenolic compound was (6)-gingerol, which is characteristic of fresh rhizomes and is responsible for their taste and aroma. Moreover, high amounts of (6)-shogaol were determined, which is interesting because this phenolic compound usually occurs in old or processed material. According to the literature, the fresh ginger rhizome either contains no shogaols or their concentrations are low [14]. Probably the spicy taste of fresh Japanese ginger is due to the high concentration of (6)-shogaol.

Sesquiterpenes represented the major source of ginger volatiles. The highest concentrations were determined for α-zingiberene, β-sesquiphellandrene, (*E*,*E*)-α-farnesene, geranial, and *ar*-curcumene. Sesquiterpene composition was similar to Chinese ginger; however, many differences were elucidated. In general, the volatiles composition of ginger cultivated on Shikoku Island is specific and strongly differs from plants cultivated in China, Nigeria, or Australia.

The ginger rhizome is a plant material with a complicated elemental composition, which is highly correlated with the area where the plant is cultivated. The present study revealed that ginger grown in ecological plantations on Shikoku Island was characterized by a high concentration of potassium and manganese and low sodium content. The elemental composition of ginger can significantly influence its medical properties because the beneficial relationships between potassium, sodium, and a high content of magnesium can increase the positive influence of ginger on the cardiovascular system. Moreover, because of its high content of potassium and manganese, ginger can be a good source of these elements in the human diet. A high content of these and of other analyzed elements, significantly higher than those previously reported in the scientific literature, can be associated with their high concentrations in the fertile soil of volcanic origin occurring in Japan. These conclusions may be supported by the results obtained by Govender and co-investigators [43], who found a significant impact of the mineral concentration in soil on the ginger “flesh” elemental composition; however, according to the authors, the plant is supposed to have its own control mechanism to regulate the metal uptake. The present study confirmed the findings obtained by Wagesho and Chandravanshi [34]: ginger may be a good source of some essential elements for humans. The elemental composition of ginger rhizomes cultivated in ecological plantations is more beneficial for human health than ginger cultivated elsewhere, as the products contain higher amounts of some macro- and trace elements and lower levels of toxic heavy metals.

## Figures and Tables

**Table 1:** Volatile components identified in the extracts from fresh ginger rhizomes.

Compound	Retention Time	Retention Index	Content (%)	Compound	Retention Time	Retention Index	Content (%)
α-Pinene	9.04	1009	3.8	Citronellyl acetate	22.29	1535	0.13
Camphene	9.61	1025	1.04	α-Copaene	22.96	1566	0.47
Myrcene	11.20	1086	0.35	Geranyl acetate	23.13	1575	0.29
α-Phellandren	11.65	1102	0.37	*cis*-β-Elemene	23.38	1586	0.83
γ-Terpinen	12.48	1133	5.12	Sesquithujene	23.71	1601	0.27
Linalool	14.89	1224	0.12	β-Ylangene	24.09	1620	0.17
Citronellal	16.55	1291	0.23	β-Copaene	24.36	1632	0.09
Borneol	16.94	1306	1.25	γ-Elemene	24.44	1637	0.79
Terpinen-4-ol	17.31	1328	0.12	(*E*)-β-Farnesene	24.99	1662	0.68
Isogeraniol	17.48	1328	0.10	allo-Aromadendrene	25.17	1671	0.23
α-Terpineol	17.72	1337	0.52	*ar*-Curcumene	25.70	1696	6.29
*n*-Decanal	18.20	1356	0.41	α-Zingiberene	26.10	1716	37.87
Citronellol	18.85	1384	0.26	(*E*,*E*)-α-Farnesene	26.34	1731	9.62
Neral	19.20	1399	1.13	γ-Cadinene	26.40	1745	0.70
Geraniol	19.70	1420	0.26	β-Sesquiphellandrene	26.76	1750	11.38
Geranial	20.10	1438	8.24	(*E*)-γ-Bisabolene	26.89	1759	0.23
Isobornyl acetate	20.49	1455	0.14	Elemol	27.31	1779	0.63
Tridecane	20.69	1464	0.25	Zingiberenol	28.25	1829	0.44
δ-Elemene	21.92	1518	0.18				
				Total content	~95%		

**Table 2:** Average content of the major phenolic compounds in fresh ginger rhizomes cultivated on ecological plantations in Japan.

Compound	(6)-Gingerol	(8)-Gingerol	(10)-Gingerol	(6)-Shogaol	(10)-Shogaols
Mean content (mg/kg)	268.3	68.8	75.2	133.2	16.0
SD	25.2	6.44	6.52	9.74	0.28
RSD (%)	9.40	9.36	8.67	7.31	1.74

**Table 3:** The average content of elements in ginger cultivated in ecological plantations on Shikoku Island (Japan) (mg/kg).

Macroelements					Trace Elements					Heavy Metals		
Elements	Ca	Mg	K	Na	Zn	Cu	Fe	Mn	Cr	Ni	Pb	Cd
Fresh rhizome	159.0 ± 31.3	239.8 ± 21.8	4052 ± 443	115.6 ± 31.4	2.54 ± 0.44	1.03 ± 0.06	12.5 ± 2.67	69.9 ± 10.0	0.064 ± 0.01	0.163 ± 0.02	0.021 ± 0.006	0.002 ± 0.0008
Dry weight	1587 ± 337	2572 ± 485	40,963 ± 9359	1261 ± 427	25.0 ± 6.90	11.1 ± 1.76	140.5 ± 49.4	758.4 ± 150.3	0.795 ± 0.054	1.67 ± 0.28	0.218 ± 0.032	0.02 ± 0.004

**Table 4:** Concentration of macro- and trace elements in the reference material.

Macro- and Trace Elements									
Parameter	Ca	Mg	K	Na	Zn	Cu	Fe	Mn	Cr
Theoretical value (mg/kg)	3522	752.3	10,260	6300	24	2.94	22.9	9.02	0.15
Found value [mg/kg]	3452	808	11,191	5743	25.7	3.55	19.7	10.6	0.15
	3609	808	12,105	5969	25.8	3.30	21.1	9.36	0.14
	3181	725	10,184	4965	22.9	3.41	21.1	9.75	0.14
	3625	731	11,441	6181	24.1	3.41	20.8	9.86	0.16
	3741	703	12,043	5281	22.8	3.47	21.8	9.54	0.17
	3618	765	11,673	5917	24.2	3.39	24.1	10.4	0.17
Average	3538	756.7	11,440	5676	24.3	3.42	21.4	9.93	0.16
SD	197.5	44.5	707.1	461.5	1.29	0.0861	1.46	0.50	0.01
RSD (%)	5.58	5.88	6.18	8.13	5.30	2.51	6.79	5.00	6.25
Recovery (%)	100.5	100.6	111.5	90.1	101.0	116.4	93.6	110.1	107
LOD (µg/kg)	180	30	60	78	41	187	154	174	0.57
LOQ (µg/kg)	600	110	250	281	152	655	513	618	2.20

**Table 5:** Concentration of heavy metals in the reference material.

Parameter	Ni	Pb	Cd
Theoretical value (mg/kg)	0.5	0.5	0.3
Found value (mg/kg)	0.51	0.52	0.34
	0.54	0.54	0.33
	0.49	0.56	0.35
	0.61	0.48	0.31
	0.56	0.55	0.34
	0.47	0.51	0.31
Average	0.53	0.53	0.33
SD	0.05	0.03	0.02
RSD (%)	9.63	5.66	6.1
Recovery (%)	106	106	110
LOD (µg/kg)	0.39	1.37	0.08
LOQ (µg/kg)	1.57	5.01	0.30

## Supplementary Materials

Supplementary materials can be found at www.mdpi.com/1422-0067/18/2/452/s1.
