# Supplementary material for: Application of Chromatographic and Spectroscopic Methods towards the Quality Assessment of Ginger (Zingiber officinale) Rhizomes from Ecological Plantations
Source: Int J Mol Sci. 2017 Feb 20;18(2):452. doi: 10.3390/ijms18020452 (PMC5343986; doi:10.3390/ijms18020452)
Supplement: Supplementary file 1 [file ijms-18-00452-s001.pdf]

Supplementary Materials: Application of Chromatographic and Spectroscopic Methods towards the Quality Assessment of Ginger (*Zingiber officinale*) Rhizomes from Ecological Plantations

Wojciech Koch, Wirginia Kukula-Koch, Zbigniew Marzec, Elwira Kasperek, Lucyna Wyszogrodzka-Koma, Wojciech Szwerc and Yoshinori Asakawa

Table S1. Concentration of standard solution [mg/L] and calibration curve parameters for FAAS analysis.

Flask Number	1	2	3	4	5	6	7	Correlation Coeff. (*R*)	Precision (% RSD)
Ca	0.5	1.0	2.0	4.0	6.0	8.0	10.0	0.9997	1.5-2.0
Mg	0.1	0.2	0.4	0.6	0.8	1.0	2.0	0.9998	1.2-1.8
K	0.2	0.4	0.6	0.8	1.0	2.0	4.0	0.9992	2.1-4.5
Na	0.2	0.4	0.6	0.8	1.0	2.0	4.0	0.9992	1.2-2.3
Zn	0.2	0.4	0.6	1.0	1.5	2.0	-	0.9999	1.4-3.2
Cu	0.5	1.0	2.0	6.0	8.0	10.0	-	0.9994	1.7-2.2
Fe	0.5	1.0	2.0	6.0	8.0	10.0	-	0.9995	1.5-2.8
Mn	0.5	1.0	2.0	6.0	8.0	10.0	-	0.9997	1.2-3.1

Table S2. Operating parameters for FAAS analysis.

Parameter	Ca	Mg	K	Na	Zn	Cu	Fe	Mn
Wavelength [nm]	422.2	285.2	766.5	589.0	213.9	324.8	248.5	279.5
Slit widths [nm]	0.5	0.5	0.5	0.2	0.5	0.5	0.2	0.2
Hollow cathode lamp power supply [mA]	5	5	10	10	5	4	10	10
Air flow [L·min^{-1}]	5	5	5	5	5	5	5	5
Acetylene flow [L·min^{-1}]	1.1	1.1	1.1	1.1	1.1	1.1	1.1	1.0
Torch height [mm]	11.0	7.0	7.0	7.0	7.0	7.0	7.0	7.0

Table S3. The validation parameters for ETAAS analysis.

Element	Line (nm)	Drying Temp. (°C)	Pyrolysis Temp. (°C)	Atomization Temp. (°C)	Conc. Range (µg/L)	Correlation Coeff. (*R*)	Precision (%RSD)
Pb	217.00	110	900	1900	0-30	0.9999	0.6-1.4
Ni	232.00	110	1050	2300	0-70	0.9999	0.8-2.2
Cr	357.87	110	1300	2300	0-10	0.9998	0.5-6.4
Cd	228.80	110	700	1600	0-2	0.9995	0.6-1.5

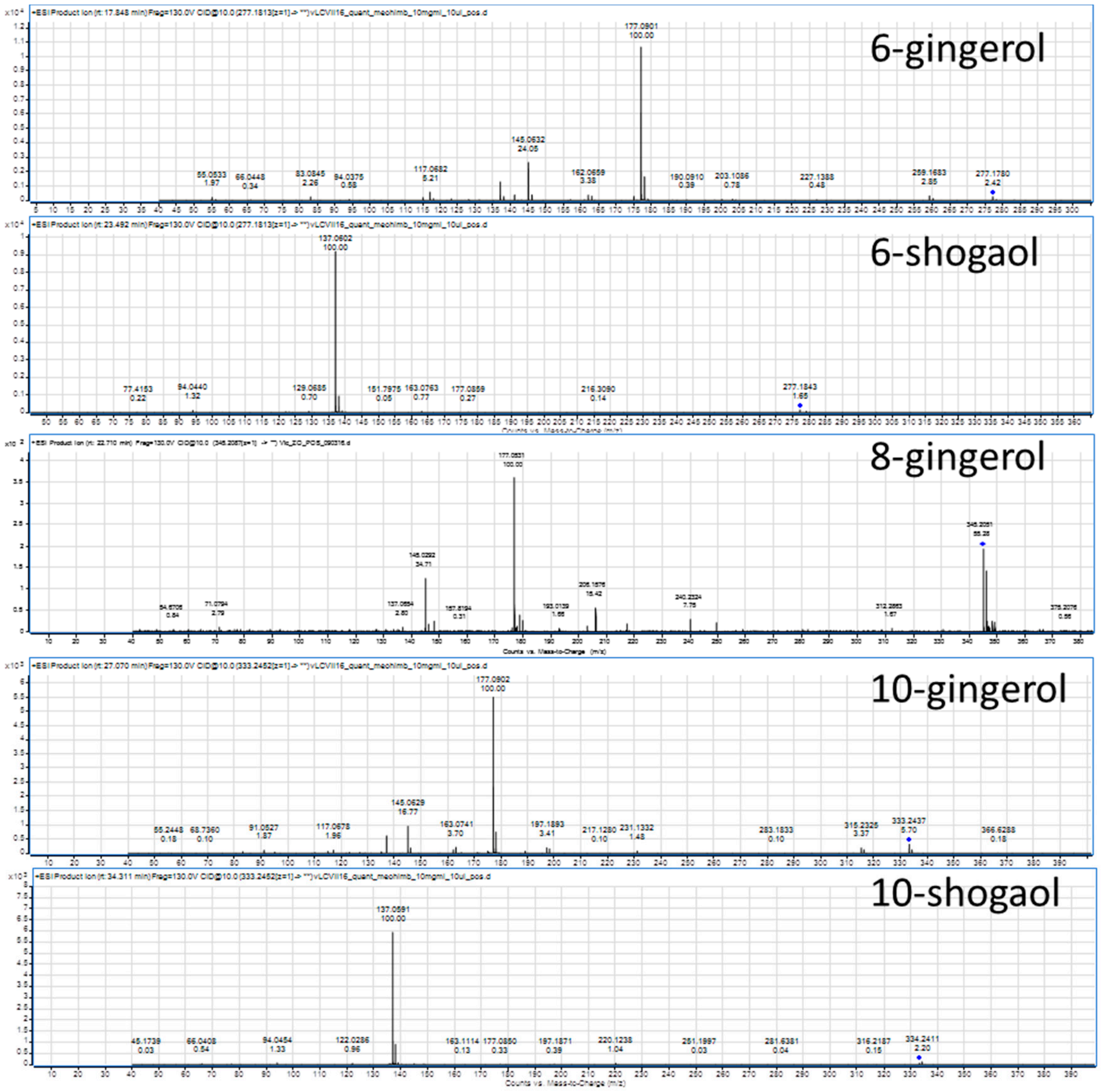

Figure S1. MS/MS spectra of gingerols and shogaols determined in the study recorded in the fragmentor energy of 130 V and CID energy of 10 V.
